# Supplementary material for: Do Rutin and Quercetin Retain Their Structure and Radical Scavenging Activity after Exposure to Radiation?
Source: Molecules. 2023 Mar 17;28(6):2713. doi: 10.3390/molecules28062713 (PMC10053567; doi:10.3390/molecules28062713)
Supplement: Supplementary file 1 [file molecules-28-02713-s001.zip › molecules-2264001-supplementary.pdf]

molecules

MDPI

Supplementary materials

Do Rutin and Quercetin Retain Their Structure and Radical Scavenging Activity after Exposure to Radiation?

Natalia Rosiak [1], Judyta Cielecka-Piontek [1], Robert Skibiński [2], Kornelia Lewandowska [3], Waldemar Bednarski [3] and Przemysław Zalewski [1,*]

[1] Department of Pharmacognosy, Poznan University of Medical Sciences, Rokietnicka 3, 60-806 Poznan, Poland
[2] Department of Medicinal Chemistry, Medical University of Lublin, Jaczewskiego 4, 20-090 Lublin, Poland
[3] Institute of Molecular Physics, Polish Academy of Sciences, Smoluchowskiego 17, 60-179 Poznań, Poland
* Correspondence: pzalewski@ump.edu.pl; Tel.: +48-(61)-854-67-10

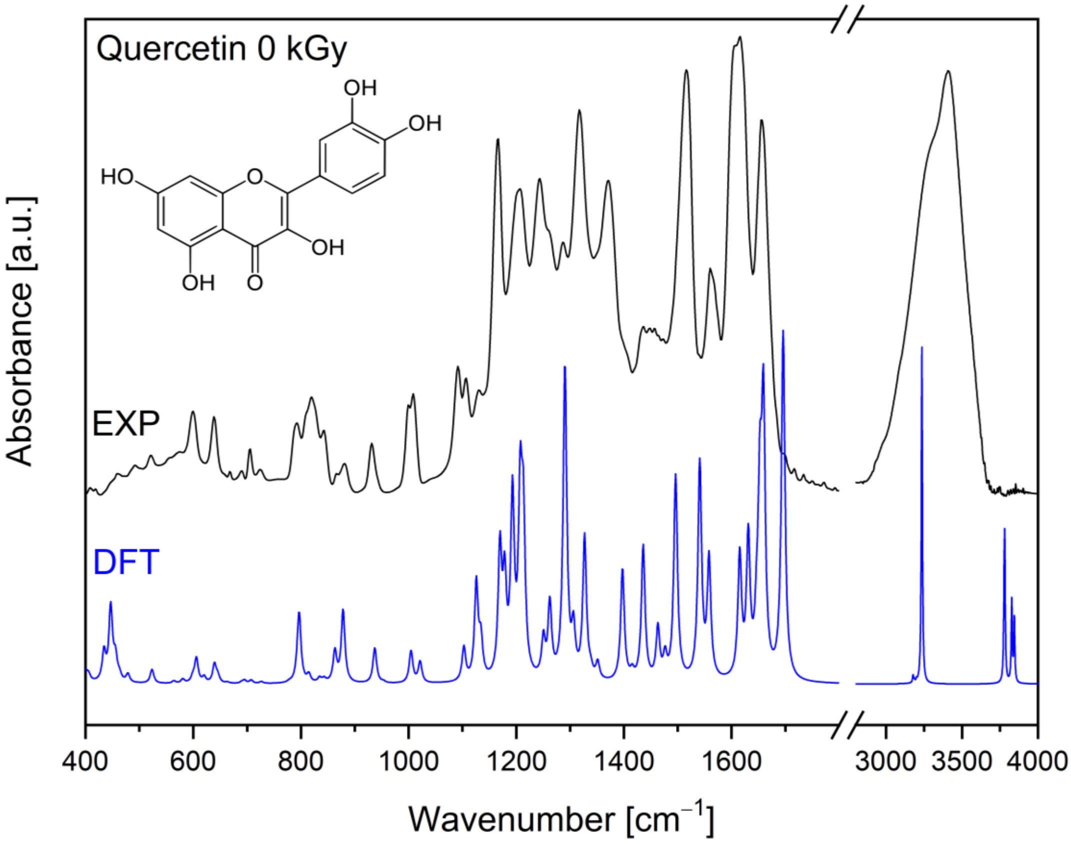

Figure S1. Theoretical (DFT, blue line) and experimental (EXP, black line) absorption spectra in IR range of quercetin.

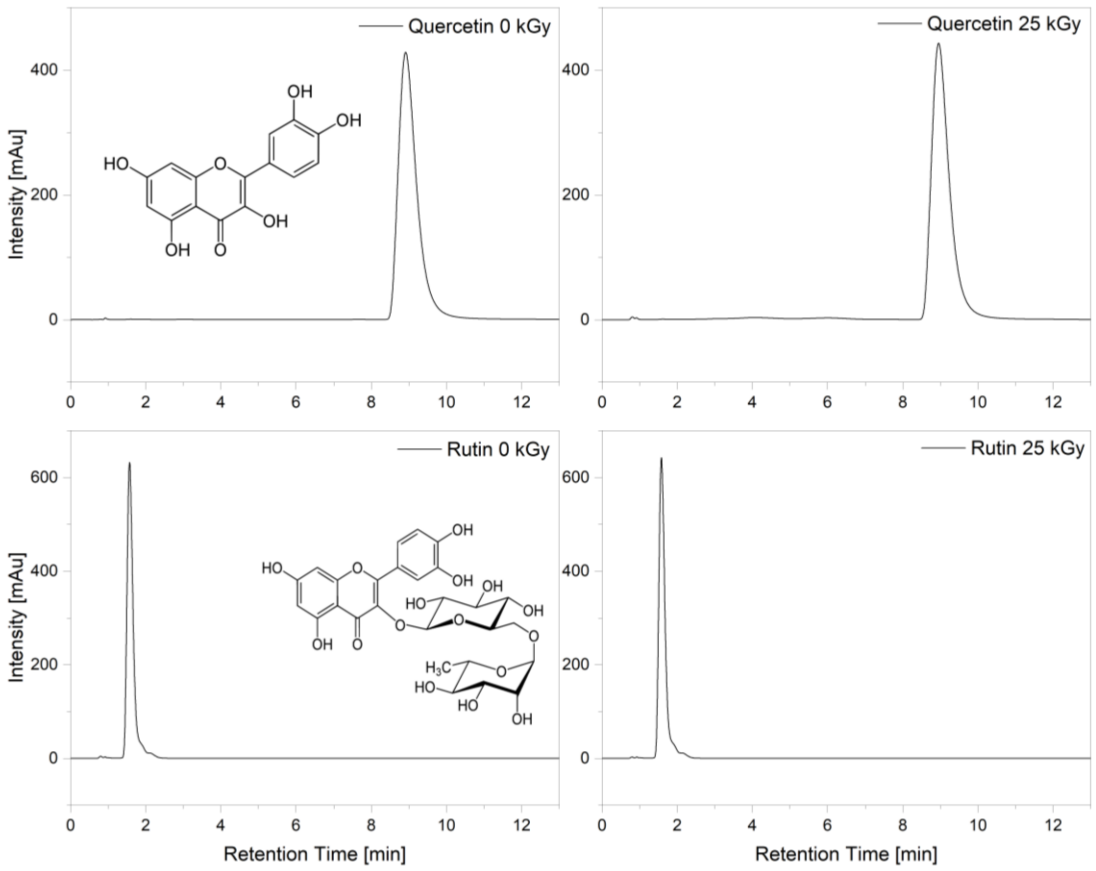

Figure S2. The HPLC analysis of non-irradiated quercetin (Quercetin 0 kGy), non-irradiated rutin (Rutin 0 kGy), irradiated quercetin (Quercetin 25 kGy), and irradiated rutin (Rutin 25 kGy).

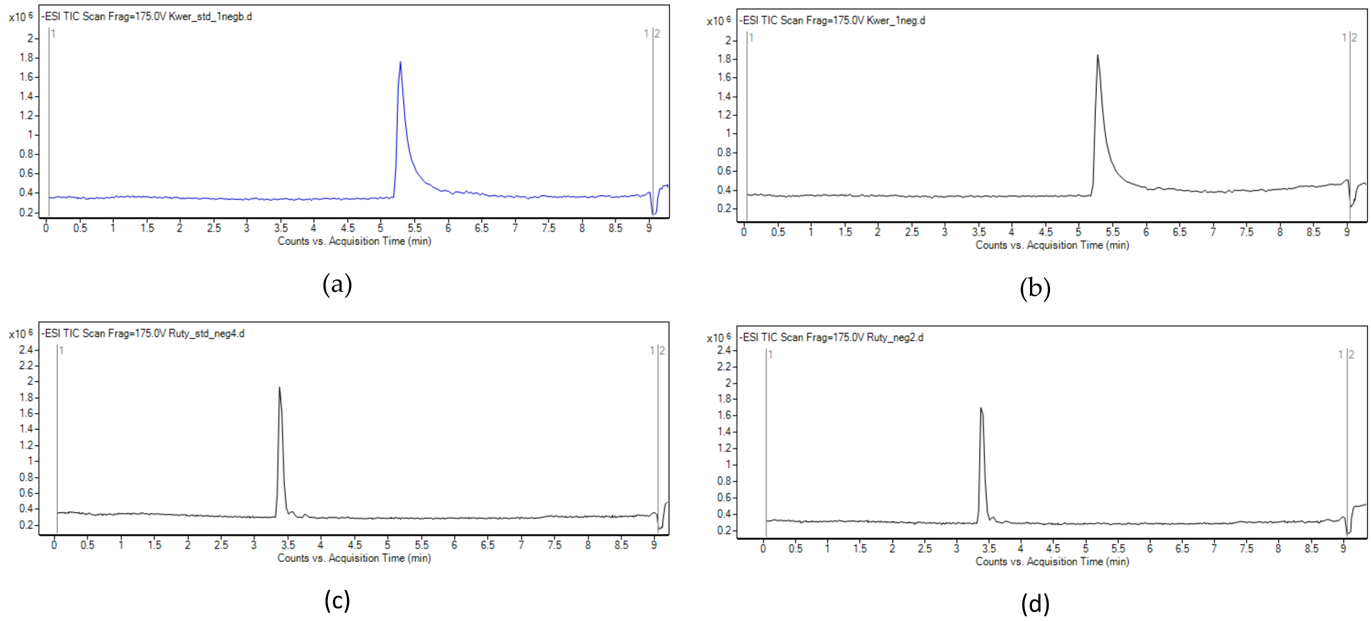

Figure S3. The LC-MS analysis: non-irradiated quercetin (a); irradiated quercetin (25 kGy) (b); non-irradiated rutin (c); irradiated rutin (25 kGy) (d).

Table S1. Selected experimental and theoretical modes (in cm−1) characteristic vibronic features of quercetin

Calculation (cm^{-1})	Experimental (cm^{-1})	Band assignment
600	598	Def. trihydroxychromenone group
640	637	Def. dihydroxyphenyl group
708	706	Def. all molecule
798	790	C–H oop at trihydroxychromenone group + breatheing dihydroxyphenyl
815	820	C–H oop at trihydroxychromenone group
879	880	O–H oop at trihydroxychromenone group
938	931	C–C–C b in dihydroxyphenyl group + rocking trihydroxychromenone group
1005	999	Def. trihydroxychromenone group + C–H r
1022	1005	C–H r
1103	1092	C–O s + C–O–H b at trihydroxychromenone group
1128	1108	C–H r + C–O–H b at dihydroxyphenyl group
1170	1166	C–H r + O–H r
1193	1206	C–O s in COH in trihydroxychromenone group + O–H r + C–H r + C–O s
1208	1244	C–H r + C–O s
1251	1262	C–O s in dihydroxyphenyl group + C–O–H b in dihydroxyphenyl group + C–C s + C–O s + C–O–H b
1263	1288	O–H r
1291	1317	C–C s + C–O s in dihydroxyphenyl group + C–O–H b + C–H r
1328	1371	C–C s between dihydroxyphenyl and trihydroxychromenone groups + C–O–H b + C–H r
1464	1434	C–O s + C–C s + C–O–H b + C=C s in dihydroxyphenyl group + C–H r
1497	1460	C–C s + C–O s + C–H r + C–O–H b
1542	1517	C–C s + C–O–H b + C–H r + C–O s in dihydroxyphenyl group
1616	1562	C=C s + C–C s + C–O–H b
1660	1616	C=O s + C–C s + C=C s + C–O s in dihydroxyphenyl group + C–H r
1697	1656	C=O s + C–O–H b + C=C s
3221	3274	C–H s
3778	3406	O–H s

Legend: b-bending, def.-deformation, oop-out of plane, r-rocking, s-stretching

Table S2. Results of HPLC-MS analysis

Comp. No.	Name	Retention time	Measured mass (*m*/*z*)	Theoretical mass (*m*/*z*)	Mass error (ppm)	Molecular ion formula [M-H$^-$]	MS/MS fragmentation ions (m/z)	MS/MS fragment formula
Quercetin								
1	Quercetin	5.3	301.0361	301.0354	2.49	$C_{15}H_9O_7$	178.9983<br>151.0036<br>121.0292<br>107.0139	$C_8H_3O_5$<br>$C_7H_3O_4$<br>$C_7H_5O_2$<br>$C_6H_3O_2$
Rutin								
Comp. No.	Name	Retention time	Measured mass (m/z)	Theoretical mass (m/z)	Mass error (ppm)	Molecular ion formula [M-H-]	MS/MS fragmentation ions (m/z)	MS/MS fragment formula
1	Rutin	3.4	609.1462	609.1461	0.2	$C_{27}H_{29}O_{16}$	343.0455<br>300.0279<br>271.0261<br>255.0310<br>151.0010	$C_{17}H_{11}O_8$<br>$C_{15}H_9O_7$<br>$C_{14}H_7O_6$<br>$C_{14}H_7O_5$<br>$C_7H_3O_4$
2	hyperin	3.54	463.0881	463.0882	0.2	$C_{21}H_{19}O_{12}$	300.0268<br>271.0247<br>255.0291<br>243.0296<br>151.0003	$C_{15}H_9O_8$<br>$C_{14}H_7O_6$<br>$C_{14}H_7O_5$<br>$C_{13}H_7O_5$<br>$C_7H_3O_4$
